# Computing interaction probabilities in signaling networks

**DOI:** 10.1186/s13637-015-0031-8

**Published:** 2015-11-11

**Authors:** Haitham Gabr, Juan Carlos Rivera-Mulia, David M. Gilbert, Tamer Kahveci

**Affiliations:** 1 Department of Computer & Information Science & Engineering, University of Florida, Gainesville, Florida, USA; 2 Department of Biological Science, Florida State University, Tallahassee, Florida, USA

**Keywords:** Biological networks, Signaling, Interaction probability, Reachability, Leukemia

## Abstract

Biological networks inherently have uncertain topologies. This arises from many factors. For instance, interactions between molecules may or may not take place under varying conditions. Genetic or epigenetic mutations may also alter biological processes like transcription or translation. This uncertainty is often modeled by associating each interaction with a probability value. Studying biological networks under this probabilistic model has already been shown to yield accurate and insightful analysis of interaction data. However, the problem of assigning accurate probability values to interactions remains unresolved. In this paper, we present a novel method for computing interaction probabilities in signaling networks based on transcription levels of genes. The transcription levels define the signal reachability probability between membrane receptors and transcription factors. Our method computes the interaction probabilities that minimize the gap between the observed and the computed signal reachability probabilities. We evaluate our method on four signaling networks from the Kyoto Encyclopedia of Genes and Genomes (KEGG). For each network, we compute its edge probabilities using the gene expression profiles for seven major leukemia subtypes. We use these values to analyze how the stress induced by different leukemia subtypes affects signaling interactions.

## Introduction

Biological networks describe how different molecules, such as proteins, interact with each other to carry out various cellular functions. Studying biological networks gives us deep insight into cellular mechanics and allows us to understand how biological processes are governed. Discovering signaling pathways [1], mapping transcription regulation [2], and identifying the reasons behind and the consequences of various disorders [3, 4] are only a few examples to many applications which are possible through studying biological networks.

Biological networks are often modeled as graphs, where each node denotes a molecule and each edge denotes an interaction. One of the critical factors that affects our analysis of biological networks is that their topologies are often uncertain. This uncertainty follows from the fact that key biological processes governing these interactions, like DNA replication, gene transcription, and epigenetic mutations, are themselves inherently uncertain events. For instance, in higher eukaryotes, DNA replication can start at different chromosome locations with different probabilities [5]. Also, different biological processes like replication timing, gene expression, and transcription regulation vary across different cell types [6–9], and also from healthy cases to different disorders [10, 11]. Probabilistic networks model this uncertainty in a mathematically sound manner. Briefly, a probabilistic biological network associates each edge of the underlying network with a probability value indicating the chance that the corresponding interaction takes place.

Taking the edge probabilities into account is extremely important in studying biological networks as they improve the accuracy of analysis of these networks and can lead to biologically significant observations that are impossible to achieve otherwise. Signaling pathway detection [1], network topology characterization [12], signal reachability [13], node centrality, and network stability [14] are just a few examples to the applications that have already been benefiting from this knowledge. Therefore, having accurate knowledge of edge probabilities is of utmost importance.

In the literature, interaction probabilities are computed in several ways. MINT [15] and STRING [16], for instance, provide a confidence value for each interaction. Confidence value of an interaction represents the level of certainty in observing that interaction. This way of probability assignment compensates for the level of noise in the experiment used to observe the interaction. However, it does not account for the inherent stochasticity of the interaction events. Sharan et al. [17] addressed this problem by utilizing features like the volume of evidence present for the interaction, gene expression correlation, and network topology to learn the edge probabilities. This strategy however accounts only for the correlation between the interacting gene products, ignoring their relations with the rest of the network. *Thus, new methods which can compute edge probabilities by taking the entire network into consideration are direly needed.*

**Contributions** In this paper, we present a novel method for computing edge probabilities for a given signaling network topology. We use end-to-end signal reachability probabilities between pairs of genes to guide our computation. While it is hard to observe the probability of each individual interaction, target reachability values are much easier to observe experimentally. Moreover, they can be observed in different cell types or under different disorders, paving way for computing phenotype-specific edge probabilities.

Correlation between the transcription levels of genes has been widely used as the primary evidence for signaling and regulation [18–22]. Here, we also use gene expression correlation as the guide for signal reachability between gene pairs. For each pair of source (i.e., membrane receptor) and target (reporter, i.e., transcription factor) genes, we compute the normalized Pearson correlation value between their gene expression levels as the *empirical* signal reachability between that pair of genes. Our method computes the probability values for all the edges so that the resulting *computed* signal reachability probabilities for all source-target pairs are as close as possible to the input empirical reachability values. Given a network with *n* edges, reachability probability can be expressed as an *n*th degree function of *n* variables [23]. Optimizing this function in an exact manner requires solving a system of *n* simultaneous derivative equations. The key challenge arises from the fact that computing the function itself has an exponential time complexity, equivalent to computing all combinations of *n* objects. This makes exact optimization impossible even for medium-sized networks. To address this challenge, we develop a two-phase strategy. The first phase is global optimization using a genetic algorithm, where we search the entire space of possible edge probability assignments to obtain a good initial probability assignment. The second phase is local optimization using hill climbing technique. Here, we optimize the initial solution we found in the first phase by gradually improving the edge probabilities one edge at a time, until no further improvement is possible. More specifically, instead of optimizing all *n* variables simultaneously, we seek to optimize the value of one variable at a time. That is, at each step, we consider only one edge probability for optimization, fixing the probability values of all other edges. We show that our method produces a result that is very close to the objective. Our experiments demonstrate that our method can compute edge probabilities with high accuracy. They also show that these probability values help in identifying specific genes and interactions that characterize major leukemia subtypes.

The rest of this paper is organized as follows. Section 2 describes the method in detail. Section 3 discusses our results. Section 4 concludes the paper.

## Method

In this section, we explain our method for computing edge probability values of a given probabilistic signaling network. Our method consists of two phases: global optimization and local optimization. Section 2.1 describes the key notation needed to understand our method and formally defines the problem. Sections 2.2 and 2.3 discuss the global and local optimization phases respectively.

### Preliminaries

Throughout the rest of this paper, we denote a probabilistic signaling network as a graph *G*=(*V, E, P*), where *V* denotes the set of nodes (i.e., genes), *E* denotes the set of directed edges (i.e., interactions), and $P: E \rightarrow \mathbb {R}\, \cap \, [\!0, 1]$ denotes the function that returns the existence probability of each edge in *E*. We also define the two sets $S \subseteq V$ and $T \subseteq V$ as the sets of source nodes (i.e., receptor genes) and target nodes (i.e., reporter genes). We define the |*S*|×|*T*| matrix *C* as the gene coexpression matrix, such that *C*[ *s,t*] is the absolute value of the Pearson correlation coefficient between the expressions of genes *s* and *t*, for all *s*∈*S* and *t*∈*T*. Given a probability function $P: E \rightarrow \mathbb {R}\, \cap \, [\!0, 1]$, we define the |*S*|×|*T*| matrix *R*_*P*_ as the signal reachability matrix, such that *R*_*P*_[ *s,t*] is the probability of a signal propagating successfully from *s* to *t*, for all *s*∈*S* and *t*∈*t* using *P*. In these definitions, *C* represents the *empirical* reachability probability between receptor and reporter genes based on their transcriptional activities. This is motivated by evidence of a strong link between gene coexpression and signaling and regulation [18–22]. On the other hand, *R*_*P*_ denotes the *computed* reachability probability between the same gene pairs, having *P* as the probability function. Thus, the Euclidean L2 norm ∥*C*−*R*_*P*_∥_2_ is the error introduced by the function *P*. Since we are only interested in the magnitude of the difference between *C* and *R*_*P*_, we used L2 norm to disregard the sign of this difference. Following from this observation, next we mathematically define the problem considered in this paper.

**Problem definition.** Given *V*, *E*, *S*, *T*, and *C*, find the function $P: E \rightarrow \mathbb {R}\, \cap \, [\!0, 1]$ such that ∥*C*−*R*_*P*_∥_2_ is minimum.

Notice that the problem above differs from the classical reachability problem. In the reachability problem, *P* is known and the goal is to find *R*_*P*_ [23]. On the other hand, in the problem considered in this paper, *P* is not known. In fact, the goal is to compute *P* with the guidance of *C*. That said, in order to understand our method in this paper, it is essential to know the original reachability problem well. In the following, we take a brief detour to summarize the PReach method that solves the reachability problem. For further details, we refer the reader to Gabr et al. [23].

Let *U*={1,…,*n*}, where *n*=|*E*|. Let *X* and *Y* be two sets of *n* variables, where *X*={*x*_1_,…,*x*_*n*_} and *Y*={*y*_1_,…,*y*_*n*_}. Let *Θ* be a subset of *U*. Let $S_{1}, \dots, S_{k}$ be *k* different subsets of *Θ*. Let $x_{S_{i}} = \prod _{j \in S_{i}}x_{j}$ and $y_{S_{i}} = \prod _{j \in S_{i}}y_{j}$, where *i*∈{1,…,*k*}. Let *x* ^∗^ and *y* ^∗^ be two free variables. Let *a*_1_,…,*a*_*k*_,*b* and *c* be real numbers. PReach defines an *xy-polynomial* over *Θ* as $F = \sum _{i=1}^{k}{a_{i}}x_{S_{i}}y_{\Theta \setminus S_{i}} + bx^{*} + cy^{*}$. Except for the free variables, each term in the above summation contains each of the indices *j*∈*Θ* either as a product term *x*_*j*_ or *y*_*j*_.

PReach associates every edge *e*_*j*_∈*E* with a variable *x*_*j*_∈*X* and a variable *y*_*j*_∈*Y*, where *j*∈*U*. In this notation, *x*_*j*_ and *y*_*j*_ represent the cases where *e*_*j*_ is present and absent, respectively. In the above summation, each of the non-free terms $\phantom {\dot {i}\!}{a_{i}}x_{S_{i}}y_{\Theta \setminus S_{i}}$ corresponds to a combination where *e*_*j*_ is present ∀*j*∈*S*_*i*_ and absent ∀*j*∈*Θ*∖*S*_*i*_, and *a*_*i*_ is the probability of observing this specific combination. The free variable *x* ^∗^ represents the case where *T* is reachable from *S*, and *b* designates its probability. Inversely, the free variable *y* ^∗^ represents the case where *T* is unreachable from *S*, and *c* designates its probability.

Let *p*_*i*_=*P*(*e*_*i*_) and *q*_*i*_=1−*p*_*i*_, ∀*e*_*i*_∈*E*. PReach starts by associating every edge *e*_*i*_∈*E* with a binomial *p*_*i*_ *x*_*i*_+*q*_*i*_ *y*_*i*_. It then proceeds by multiplying these binomials together into a growing *xy*-polynomial. After each multiplication step, PReach checks the polynomial for the non-free terms that can be *collapsed* into one of the two free terms as follows. For any of the non-free terms $\phantom {\dot {i}\!}{a_{i}}x_{S_{i}}y_{\Theta \setminus S_{i}}$, if the edges associated with *S*_*i*_ form at least one path from *S* to *T*, it replaces those terms with *a*_*i*_ *x* ^∗^. Inversely, if the edges associated with *Θ*∖*S*_*i*_ form at least one cut between *S* and *T*, it replaces those terms with *a*_*i*_ *y* ^∗^. Any further multiplication of a new term *p*_*i*_ *x*_*i*_ with *b* *x* ^∗^ results in *b* *p*_*i*_ *x* ^∗^. Similarly, (*p*_*i*_ *x*_*i*_)(*c* *y* ^∗^)=*c* *p*_*i*_ *y* ^∗^, (*q*_*i*_ *y*_*i*_)(*b* *x* ^∗^)=*b* *q*_*i*_ *x* ^∗^, and (*q*_*i*_ *y*_*i*_)(*c* *y* ^∗^)=*c* *q*_*i*_ *y* ^∗^. The reachability problem is a computationally hard problem; it belongs to the #P-complete class [24]. However, thanks to the repeated application of the collapsing operation, PReach tries to avoid exponential growth of the size of the *xy*-polynomial.

### Phase 1: global optimization

We are now ready to describe the method developed in this paper. The first phase of our method is a genetic algorithm to find a population of probability functions as an initial candidate solution. Note that this is a best-faith solution that will be further optimized in the second phase of our method.

We represent a candidate solution as a vector with |*E*| entries and denote it with *ψ*, where the *i*th entry *ψ*[ *i*] is the probability assigned to edge *e*_*i*_∈*E*. Let us denote the computed reachability matrix obtained using the solution *ψ* as *R*_*ψ*_. That is, ∀*s*∈*S*, ∀*t*∈*T*, *R*_*ψ*_[ *s,t*] is the signal reachability probability between *s* and *t*, computed based on edge probabilities in *ψ* (see Section 2.1). We define the fitness *F*_*ψ*_ of a candidate solution *ψ* as $1 - \frac {\|C-R_{\psi }\|_{2}}{|S \times T|}$. In this formulation, the fitness *F*_*ψ*_ takes a value in the [ 0,1] interval. A larger value indicates a better solution. In the extreme case when the empirical and computed reachability probabilities are identical (i.e., *C*=*R*_*ψ*_), *F*_*ψ*_ is equal to 1, indicating that the solution is 100 *%* accurate.

Our genetic algorithm consists of four steps: initialization, crossover, mutation, and selection. We elaborate on these steps next.

- **Initialization** We start by generating a set *Ψ* of random candidate solutions. These solutions serve as the seed population of solutions. We generate each seed candidate solution *ψ*∈*Ψ* by assigning a random number between 0 and 1 to each entry in *ψ*. We then compute the fitness values *F*_*ψ*_, ∀*ψ*∈*Ψ*. In our experiments, we set the population size to |*Ψ*|=50, thus generate 50 random seeds.
- **Crossover** This step improves the solutions in the set *Ψ* by combining pairs of existing solutions, also known as the crossover operation. To do that, We define a *gap* value *g*_*ψ*_ for every *ψ*∈*Ψ* as $g_{\psi } = \sum _{i=1}^{|S|} \sum _{j=1}^{|T|} C[\!i,j] - R_{\psi }[\!i,j]$. The value of *g*_*ψ*_ shows how much the reachability *R*_*ψ*_, computed based on the solution *ψ*, deviates from the target *C* in total. A positive gap value indicates that the solution *ψ* underestimates the probability of some of the edges. Inversely, a negative gap value indicates that the solution *ψ* overestimates the probability of some of the edges. We then randomly select two solutions *ψ*_1_ and *ψ*_2_ from *Ψ* using biased sampling, where the chance of selecting a sample *ψ*_*i*_ is directly proportional to its fitness $F_{\psi _{i}}$. We use *ψ*_1_ and *ψ*_2_ to generate a new candidate solution as follows. For each entry *i*∈{1,..,|*E*|}, we choose either entry *ψ*_1_[ *i*] or *ψ*_2_[ *i*] based on which is more likely to produce a candidate solution with a higher fitness. There are three possible scenarios: if both $g_{\psi _{1}}$ and $g_{\psi _{2}}$ are positive, both *ψ*_1_[ *i*] and *ψ*_2_[ *i*] are possibly underestimated, so we choose the higher. Inversely, if both $g_{\psi _{1}}$ and $g_{\psi _{2}}$ are negative, both *ψ*_1_[ *i*] and *ψ*_2_[ *i*] are possibly overestimated, so we choose the lower. If one of $g_{\psi _{1}}$ and $g_{\psi _{2}}$ is positive and the other is negative, then we randomly select between *ψ*_1_[ *i*] and *ψ*_2_[ *i*], where the chance of each is proportional to the fitness of its corresponding solution. We expect this strategy to produce a new solution that is better than both *ψ*_1_ and *ψ*_2_, as we reduce the gap value while constructing it. We repeat the crossover step 50 times (i.e., |*Ψ*| times) and include the resulting solutions to *Ψ*.
- **Mutation** In this step, our genetic algorithm aims to avoid local minima by adding a small amount of random diversity to the existing set of solutions. More specifically, for each solution *ψ*∈*Ψ*, we iterate over all entries *ψ*[ *i*]. For each entry *ψ*[ *i*], we perform a Bernoulli trial with probability of 0.01. If the trial yields success, the entry value is replaced with a new value drawn uniformly at random from the range [0, 1].
- **Selection** After crossover and mutation, the size of *Ψ* doubles to 100. This step ensures the set of solutions in *Ψ* does not grow. To do this, from the 100 solutions in *Ψ*, we select five which have the highest fitness values. Additionally, we randomly select another 45 solutions from the remaining 95, where every solution has a chance of selection that is proportional to its fitness. We remove the non-selected 50 solutions from *Ψ*.

We repeat the crossover, mutation, and selection steps for a large number of iterations, updating the population *Ψ* each time. The number of iterations needed for convergence depends on the size and properties of the target network and is a matter of trial and error. We then select the solution which has the highest fitness in the final population as the output of this phase. We use it as an input to our next local optimization phase.

### Phase 2: local optimization

At the end of the first phase, we have a solution *ψ* that has the highest fitness value in the entire population *Ψ*. Although *ψ* is expected to yield small errors in signal reachability, it is not necessarily optimal. In this phase, we develop a hill climbing algorithm, which gradually alters the probability assignment of each edge in the solution, one edge at a time. At each step, it ensures that the probability assignment *ψ*[ *e*] of the edge *e* being altered becomes optimal (i.e., yields the highest possible fitness value) given the probability assignment of all other edges. We continue altering the solution until no edge probability value can be altered without increasing ∥*C*−*R*_*ψ*_∥_2_. In the following, we describe in detail how at a given step we alter one probability assignment *ψ*[ *e*], given all other values in *ψ*.

**Optimizing a single edge probability** Assume that for only one edge *e*∈*E*, the probability *p*_*e*_ of this edge is unknown. Also, assume that the probability values of all the remaining edges in *E*−{*e*} are known. Here, we compute the value of *p*_*e*_ that guarantees to minimize the reachability error ∥*C*−*R*_*ψ*_∥_2_. For this purpose, we develop a new method which is built on the PReach method [23].

Unlike PReach, our method allows one of the edge probabilities *p*_*e*_ to be a variable. This additional unknown alters the form of the *xy*-polynomial constructed by PReach (see Section 2.1) as the unknown *p*_*e*_ can get multiplied by all the terms of the original *xy*-polynomial. This new variable can increase the polynomial size dramatically, depending on the combination of the terms in the polynomial. We avoid this problem through a simple observation that the final *xy*-polynomial is independent of the order in which we multiply individual edge binomials. Following from this observation, we defer the multiplication of the edge binomial corresponding to *e* until all other edge binomials are multiplied. Thus, until before the edge binomial of *e* is multiplied, our method yields the same intermediate *xy*-polynomial as PReach. After multiplying the final binomial by the intermediate *xy*-polynomial, the coefficient of *x* ^∗^ in the final *xy*-polynomial has the form *α*+*β* *p*_*e*_, where *α* and *β* are real numbers. We mathematically deduce the values of *α* and *β* in the following theorem.

#### **Theorem1**.

Assume the binomials of all edges except that of *e* has been already multiplied into the *xy*-polynomial $F = \sum _{i=1}^{l}{a_{i}}x_{S_{i}}y_{\Theta \setminus S_{i}} + bx^{*} + cy^{*}$, where the terms $\phantom {\dot {i}\!}{a_{i}}x_{S_{i}}y_{\Theta \setminus S_{i}} \forall i \in \{1, \ldots, l\}$ are *l* terms that are not yet collapsed into either *x* ^∗^ or *y* ^∗^. The reachability probability, which is the final coefficient of *x* ^∗^ after multiplying the binomial of *e*, is *α*+*β* *p*_*e*_, where *α*=*b* and $\beta = \sum _{i=1}^{l}{a_{i}}$.

#### *Proof*.

Multiplying the *e* binomial (*p*_*e*_ *x*_*e*_+(1−*p*_*e*_)*y*_*e*_) by *F*, the final *xy*-polynomial *F*_final_ has the following form.

$$\begin{aligned} F_{\text{final}} &=\\ &\left(p_{e} x_{e} + (1-p_{e}) y_{e} \right)\! \left(\sum_{i=1}^{l}{a_{i}}x_{S_{i}}y_{\Theta \setminus S_{i}} + bx^{*} + cy^{*} \right)\\ &=~p_{e} x_{e} \sum_{i=1}^{l}{a_{i}}x_{S_{i}}y_{\Theta \setminus S_{i}} + (1-p_{e}) y_{e} \sum_{i=1}^{l}{a_{i}}x_{S_{i}}y_{\Theta \setminus S_{i}}\\ &\quad+~p_{e} b x_{e} x^{*} + (1-p_{e}) b y_{e} x^{*}\\ &\quad+ p_{e} c x_{e} y^{*} + (1-p_{e}) c y_{e} y^{*} \end{aligned} $$

Since *e* is the last edge to multiply in the network, it is guaranteed that $\phantom {\dot {i}\!}x_{e} x_{S_{i}}$ and $\phantom {\dot {i}\!}y_{e} y_{\Theta \setminus S_{i}}$ will collapse to *x*∗ and *y* ^∗^, respectively, for all *i*∈{1,…,*l*} [23]. Also, we already know that *x*_*e*_ *x* ^∗^=*x* ^∗^ and *y*_*e*_ *y* ^∗^=*y* ^∗^ for any edge *e*. Thus, we have

$$\begin{aligned} F_{final} &=\\ &p_{e} \sum_{i=1}^{l}{a_{i}} x^{*} + (1-p_{e}) \sum_{i=1}^{l}{a_{i}} y^{*}\\ &\quad+~p_{e} b x^{*} + (1-p_{e}) b x^{*} + p_{e} c y^{*} + (1-p_{e}) c y^{*}\\ &=\left(p_{e} \sum_{i=1}^{l}{a_{i}} + p_{e} b + (1-p_{e}) b \right) x^{*}\\ &\quad+~\left((1-p_{e}) \sum_{i=1}^{l}{a_{i}} + p_{e} c + (1-p_{e}) c \right) y^{*}\\ &=~\left(\!p_{e}\! \sum_{i=1}^{l}{a_{i}}\! +\! b\! \right) x^{*} +\left((1-p_{e}) \sum_{i=1}^{l}{a_{i}} + c \right) y^{*} \end{aligned} $$

The reachability probability is the final coefficient of *x* ^∗^. Therefore its value is $p_{e} \sum _{i=1}^{l}{a_{i}} + b$. i.e., *α*=*b* and $\beta = \sum _{i=1}^{l}{a_{i}}$. This means that after multiplying the binomials of all the edges except *e*, *α* is the coefficient of *x* ^∗^, and *β* is the sum of the coefficients of all non-free terms.

Notice that the coefficient of *x* ^∗^ (i.e., *α*+*β* *p*_*e*_) is also a polynomial of first degree in *p*_*e*_. Using this observation, we solve for the objective of this paper (which is to minimize ∥*C*−*R*_*ψ*_∥_2_) by solving for *p*_*e*_ as follows. We first compute *R*_*ψ*_[ *s,t*]=*α*_*st*_+*β*_*st*_ *p*_*e*_, ∀(*s,t*)∈*S*×*T*. We then derive the optimal value for *p*_*e*_ as:

$$\begin{array}{*{20}l} \text{Minimize}~~~&\|C-R_{\psi}\|_{2}\\ &= \sum_{(\text{\textit{s,t}}) \in S \times T} \left(C[\!s, t] - R_{\psi}[\!s, t] \right)^{2}\\ &= \sum_{(\text{\textit{s,t}}) \in S \times T} \left(C[\!s, t] - \alpha_{st} - \beta_{st} p_{e} \right)^{2}\\ \end{array} $$

To solve the minimization function above, we equate its derivative to zero.

$$\begin{array}{*{20}l} &\frac{d}{dp_{e}} \sum_{(\text{\textit{s,t}}) \in S \times T} \left(C[\!s, t] - \alpha_{st} - \beta_{st} p_{e} \right)^{2} = 0\\ &\therefore \sum_{(\text{\textit{s,t}}) \in S \times T} -2\beta_{st} \left(C[\!s, t] - \alpha_{st} - \beta_{st} p_{e} \right) = 0\\ &\therefore \sum_{(\text{\textit{s,t}}) \in S \times T} -2\beta_{st} \left(C[\!s, t] - \alpha_{st} \right) + p_{e} \sum_{(\text{\textit{s,t}}) \in S \times T} 2\beta_{st}^{2}\\ & = 0 \end{array} $$

Thus, we get

$$\begin{array}{*{20}l} p_{e} = \frac{\sum_{(\text{\textit{s,t}}) \in S \times T} \beta_{st} \left(C[\!s, t] -\, \alpha_{st} \right) }{\sum_{(\text{\textit{s,t}}) \in S \times T} \beta_{st}^{2}} \end{array} $$

The formula above constitutes the optimal value for *p*_*e*_ given all other probability values. However, there is no guarantee that optimal value of *p*_*e*_ falls within the proper probability range of [ 0,1]. This is because the derivation above gives the optimal result across all real numbers. However, by taking the second derivative of the objective function, one can easily see that the objective function is continuous, convex, and has only one solution to $\frac {d}{dp_{e}} = 0$. This implies that the closer the value of *p*_*e*_ is to its unconstrained optimal value, the smaller the error is in the objective function. Therefore, if the optimal value of *p*_*e*_ is above 1, we replace it with 1. If it is below 0, we replace it with 0. This way, we find the best possible value for *p*_*e*_ in [ 0,1].

## Experimental results

In this section, we experimentally evaluate our method on four major signaling networks from the Kyoto Encyclopedia of Genes and Genomes (KEGG), including cell cycle, programmed cell death, and immune response regulation pathways (see Table 1 for dataset details). We use the gene expression samples for the leukemia subtypes from Zhang et al. [10]. This dataset contains gene expression values for 413 patients, each having one of seven different leukemia subtypes (six B-ALL subtypes plus T-ALL). We use this dataset as it provides a large number of samples for a wide spectrum of leukemia subtypes. We perform a comprehensive comparative analysis of how interaction probabilities vary across these leukemia subtypes (Section 3.2). Using the interaction probability values we find, we also compute gene centrality values for all network and leukemia subtype combinations (Section 3.3). We finally extract the genes which behave differently in specific combinations of network and leukemia subtype, and analyze the significance of these genes in these combinations (Section 3.4).

**Table 1:** Networks used in our experiments, their sizes (nodes and edges), running time of our method to compute their interaction probabilities, and the quality of the resulting probabilities. For every network, time is the average running time over the seven leukemia subtypes in seconds, and quality is the average result quality over the seven leukemia subtypes

Network	Nodes	Edges	Time (s)	Quality (%)
Apoptosis	48	58	77.78	95.37
Cell cycle	66	79	274.78	96.08
Complement and coagulation	57	67	126.57	96.88
Chemokine	51	62	302.86	95.4

### Comparison with logistic regression

In this section, we present comparative analysis of the results of our method against the logistic regression method presented by Sharan et al. [17]. Throughout this section, we refer to our method as PReach, and to the logistic regression method as LogReg. LogReg learns interaction probabilities through three features: available evidence, interactor small-world properties, and their gene expression. The latter is different across the leukemia subtypes, therefore LogReg produces different probability values for each subtype. In addition to the four networks described in Table 1, we also use four more networks to obtain more conclusive results. The additional four networks are ErbB, Wnt, NF-kappaB, and p53 from KEGG.

**Fig. 1:**
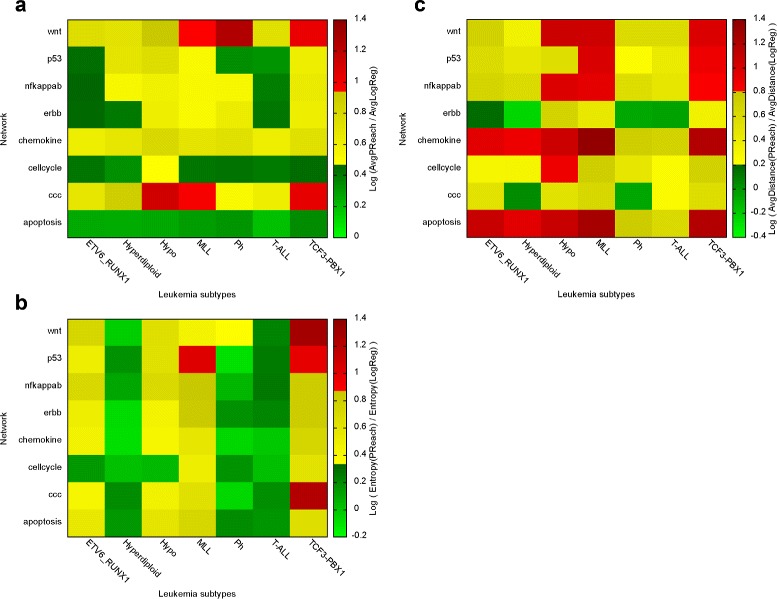
Comparison of PReach vs LogReg for computing edge probabilities in different leukemia subtypes. *X*-axis: leukemia subtypes. *Y*-axis: networks. All values are computed for both methods and compared using logarithm of the ratio (i.e., more than zero means PReach is higher). **a** Average edge probability. **b** Entropy of edge probability distribution. **c** Average distance between the edge probabilities of a each subtype and other subtypes

First, we compare the edge probability values produced by both methods. For each network, we run both methods once for each leukemia subtype. For every pair of network and subtype, we compute the average edge probability for both methods. We then compute the log of the ratio between them for comparison. Figure 1 a shows the results. We observe from the figure that PReach produces higher probabilities on average for all pairs of network and subtypes. The biggest gap between PReach and LogReg occurs in Wnt and complement and coagulation cascades (ccc).

Next, we compare the quality of the results of PReach vs LogReg. There exists no ground truth to compare the edge probability values against. However, we compare the outputs of the two methods with respect to two measures. First, we inspect how spread is the output probabilities across the [ 0,1] spectrum. To do this, we divide the [ 0,1] range into a set of ten bins *B*={[ 0,0.1),[ 0.1,0.2),…,[ 0.9,1]}, and count the number of times a probability appears in each bin. For every pair of network and subtype, we compute the entropy of the method as $ - \sum _{b \in B} p(b) log p(b)$, where *p*(*b*) is the number of values in the bin *b* divided by the total number of values. These entropy values are higher if the values are more spread across the [ 0,1] spectrum, and lower if they are crowded in a less fraction of the spectrum. Figure 1 b shows the results. We observe that the entropy in PReach is higher than LogReg in almost all cases. This means that PReach output probabilities are more spread across the [ 0,1] spectrum, while those of LogReg tend to be more discrete. More detailed inspection reveals that this happens because LogReg assigns similar probability values to most of the interactions most of the time (results not shown due to space limit). Thus, it fails to provide fine-grained distinction between the likelihoods of interactions, while our method successfully provides such distinction.

To further investigate the results quality of both methods, we inspect how much each method differentiates leukemia subtypes with respect to their edge probability values. For every pair of network and subtype, we arrange the edge probability values produced by each method in a vector with the same ordering. Next, for a given network, we compute the Euclidean distance between the vectors of each pair of leukemia subtypes. Then for every subtype, we compute the average distance between its vector and those of all other subtypes. We compare this average distance when computed using PReach versus LogReg. Figure 1 c shows the results. We observe that the distances computed based on PReach are higher in the vast majority of network-subtype pairs. This means that our method can differentiate leukemia subtypes while LogReg fails to do that.

### Interaction probability in leukemia

In this experiment, we explore the differences on interaction probabilities of signaling networks in distinct leukemia subtypes. Our aim is to identify specific gene interaction differences between distinct leukemia subtypes. To achieve this, we use our method to compute interaction probability values for the KEGG signaling networks. For each network, we run our method seven times, once for each leukemia subtype. Before conducting detailed analysis, however, we first need to validate that our method is computationally feasible, that it scales to networks under consideration. To evaluate its performance, we measure the time our method takes to compute the probabilities of all the interactions for every network in every leukemia subtype. Also, based on our original optimization target (see Section 2.1), we need to know how accurate our results are (i.e., how close the computed reachability probability *R*_*ψ*_ is to the input *C*). To do this, we measure the quality of the resulting interaction probabilities as $1 - \frac {\|C-R_{\psi }\|}{|S \times T|}$. The closer this value is to 100 %, the better the quality is.

Table 1 shows the size of each network along with its average time and quality over the seven leukemia subtypes. Our results demonstrate that our method easily scales to the networks under consideration. It computes the interaction probabilities in about 5 min or less for all the networks we tested. More importantly, our method is highly accurate. The computed reachability values deviate from the empirical reachability values by less than 5 *%* for all the networks. These results are highly encouraging as they show that our method is both accurate and has practical running time. Thus it can be applied on real datasets to compute interaction probabilities.

**Fig. 2:**
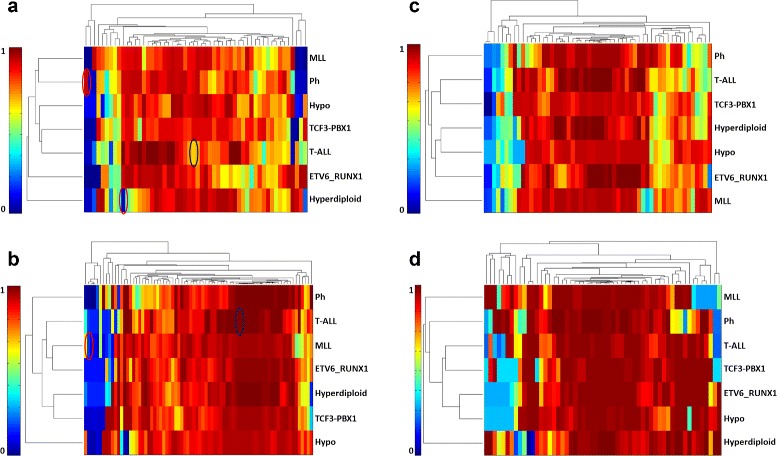
Interaction probability values of the four KEGG networks in seven leukemia subtypes. **a** Apoptosis, **b** cell cycle, **c** complement and coagulation, and (**d**) chemokine. Rows represent leukemia subtypes, and columns represent network interactions. Both rows and columns are hierarchically clustered

Next, we analyze the differences in interaction probabilities across leukemia subtypes. For each network, we represent each leukemia subtype by a vector of the edge probabilities computed for it. We then compute a hierarchical clustering of these vectors. Figure 2 shows the results. From the figure, we observe that the probability value of some interactions vary significantly across different leukemia subtypes. For instance, CASP3 is the target in three different apoptosis interactions whose probability in a subtype is at least 2 standard deviations away from their mean values among other subtypes. These interactions are (CASP10 $\rightarrow $ CASP3) in hyperdiploid, (CASP12 $\rightarrow $ CASP3) in T-ALL, and (BIRC8 $\rightarrow $ CASP3) in Ph (circled in Fig. 2 a). Similarly, CHEK1 is the source in two different cell cycle interactions whose probability in a subtype is at least 2 standard deviations away from their mean values among other subtypes. These interactions are (CHEK1 $\rightarrow $ CDC25A) in T-ALL, and (CHEK1 $\rightarrow $ TP53) in MLL (circled in Fig. 2 b). CASP3 is already linked to B-cell lymphoma [25], lung [26], skin [27], breast [28], and other cancers. Defects in apoptosis signaling and cell cycle pathways play an essential role in leukemogenesis. CASP3 is an effector caspase that has been associated with B-cell lymphoma [25], lung [26], skin [27], breast [28], and other cancers. Moreover, regulation of CASP3 activation has been linked to the prognosis and remission in B-ALL [29]. Notably, in the three leukemia subtypes with different apoptotic signals targeting CASP3, the programmed cell death is inhibited; interactions of CASP3 with its activators are weaker in hyperdiploid and T-ALL (CASP10 $\rightarrow $ CASP3 and CASP12 $\rightarrow $ CASP3, respectively) while the interaction with its inhibitor is increased in B-ALL with Philadelphia chromosome (BIRC8 $\rightarrow $ CASP3). CHEK1 is a cell cycle checkpoint response protein that is linked to oral squamous cell carcinoma [30] and colorectal cancer [31]. Recently, increased levels of CHEK1 have been associated to B-ALL and T-ALL [32]. Our observation makes both CASP3 and CHEK1 strong candidates for investigation in their respective subtypes of leukemia.

Additionally, the hierarchy of the leukemia subtypes gives an insight about which subtypes have similar signaling behavior. T-ALL and TCF3-PBX1 are closest to each other in apoptosis, complement and coagulation, and chemokine, noticeably more distant in cell cycle. Hyperdiploid is very similar to TCF3-PBX1 in cell cycle, but more distant from it in the other three networks. In fact, hyperdiploid is the most distant from all other subtypes in both apoptosis and chemokine. This information can guide us to build on the existing knowledge about signaling behavior in a certain subtype, using appropriate experiments, to develop new findings about other subtypes with similar behavior.

### Gene centrality for leukemia subtypes

**Fig. 3:**
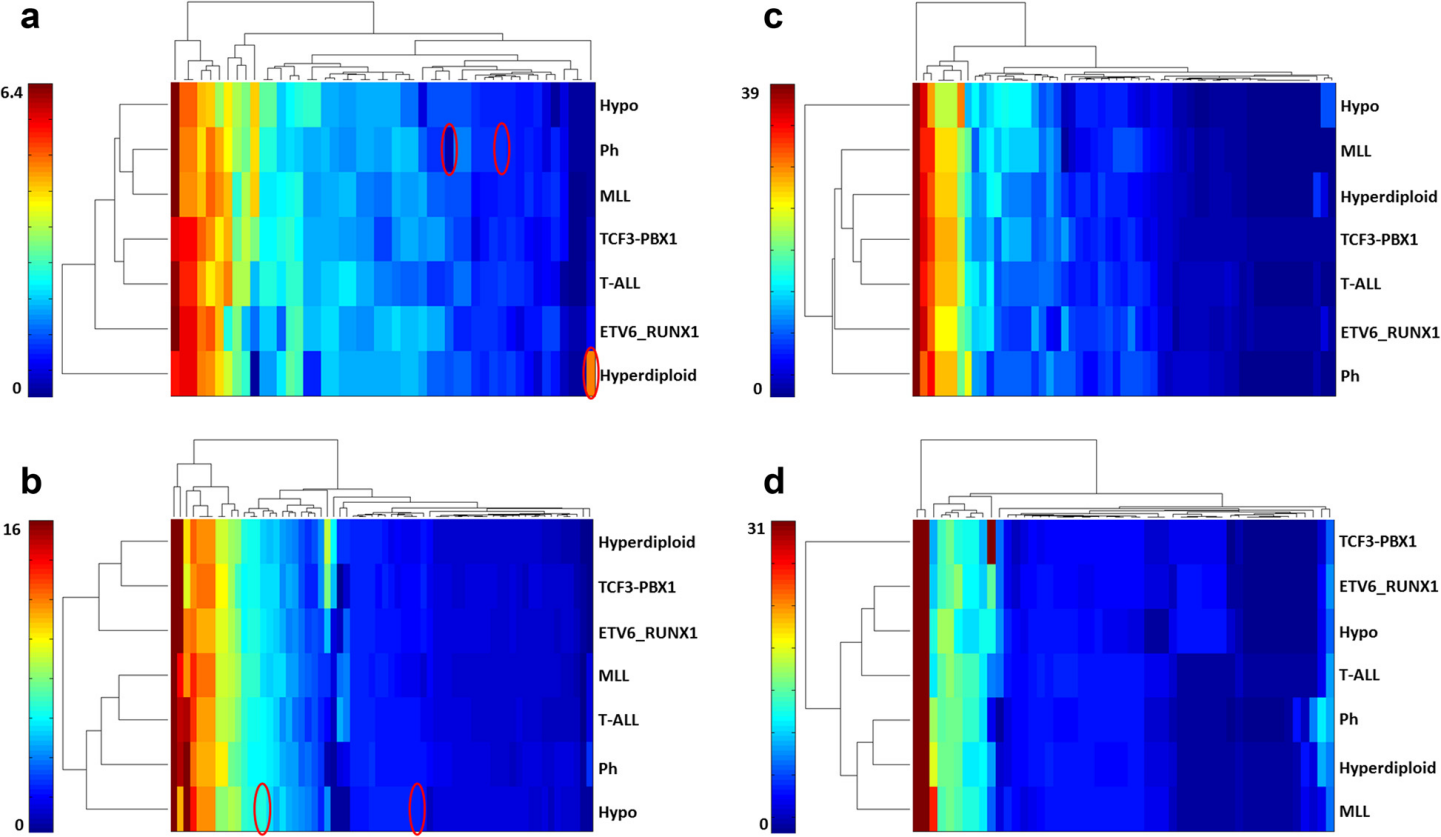
Gene centrality values of the four KEGG networks in seven leukemia subtypes. **a** Apoptosis, **b** cell cycle, **c** complement and coagulation, and (**d**) chemokine. *Rows* represent leukemia subtypes, and *columns*represent genes. Both *rows* and *columns* are hierarchically clustered

Next, we use the interaction probability values we computed in Section 3.2 to compute centrality values of the genes in each network. Briefly, we compute the centrality of a gene as its contribution to signal reachability probability between all pairs of genes (see Gabr et al. [14] for details). We compute centrality values for the genes in each of the four networks for each of the seven leukemia subtypes. For each network, we represent each leukemia subtype by a vector of the node centrality values computed for it. We then compute a hierarchical clustering of these vectors. Figure 3 presents the results.

Interestingly, the figure shows variation of centrality value across different leukemia subtypes for only a small number of genes. Notice that this variation is not as diverse as that of interaction probability values (see Fig. 2). In apoptosis, BID is the top outstanding gene in hyperdiploid, with centrality values 2.8 standard deviations higher than their mean centrality in other subtypes (circled in Fig. 3 a). We notice loss of centrality for other key regulators of apoptosis like RIPK1 and CASP7 in ETV6_RUNX1 and Ph, respectively (circled in Fig. 3 a). Similarly, cell cycle regulators like CDK1 and PLK1 have an increased centrality in hypo, with centrality values 2.6 standard deviations higher than their mean centrality in other subtypes (circled in Fig. 3 b). BID remains as key regulator in hyperdiploid but its centrality is lost in other samples, suggesting a disruption of the programmed cell death regulation in most of the leukemia subtypes. RIPK1 and CASP7 are linked to colorectal cancer [33]. CDK1 and PLK1 induce cell cycle progression and have been associated with distinct types of cancer [34–36] including leukemia and lymphoma [37–39]. Our results suggest that these genes are interesting targets for studying in the scope of their respective leukemia subtypes.

### Enrichment analysis of outstanding genes

**Fig. 4:**
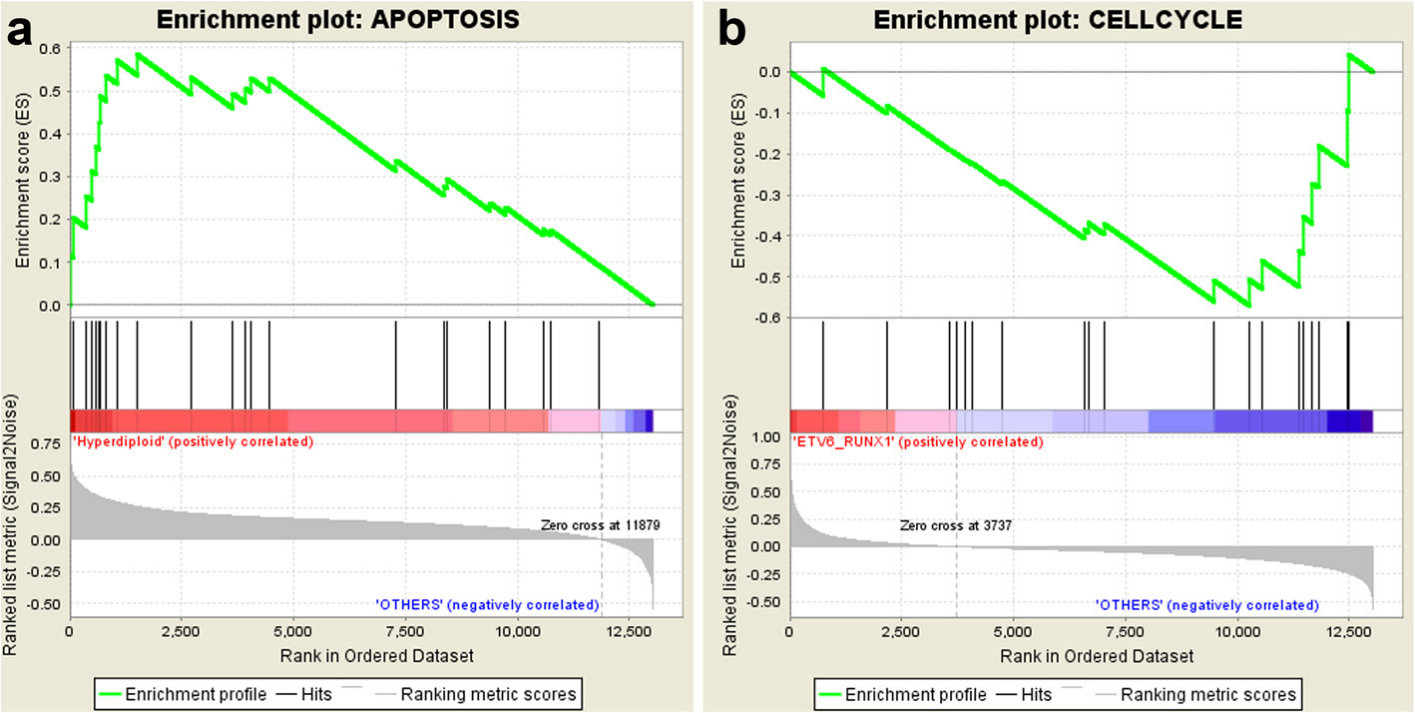
Gene set enrichment results for the highest two enriched gene sets in their respective leukemia subtypes. **a** Apoptosis in hyperdiploid and (**b**) cell cycle in ETV6_RUNX1

Following from the previous results, we want to know which network plays a key role in a certain leukemia subtype. In other words, we want to know which network’s outstanding set of genes is highly enriched in a specific leukemia subtype. To achieve this, we first extract the set *L* of outstanding genes for every network in every subtype. For every edge *e*=(*u,v*) in a given network, we compute the mean *μ*_*e*_ and the standard deviation *σ*_*e*_ of its probability values in all leukemia subtypes. Then for every subtype, for every edge *e*, we check if the probability of *e* in this subtype was at least 2*σ*_*e*_ away from *μ*_*e*_. If it is, we add *u* and *v* to *L*. We then perform gene set enrichment analysis (GSEA) [40] on *L* for every network in every leukemia subtype. For every pair of network and subtype, we set the phenotype *A* as the subtype samples, and phenotype *B* as all samples from other subtypes. We then run GSEA on the network’s outstanding gene set *L* to measure its differential significance from *A* to *B*. We consider gene sets whose *p* value is below 0.1 as highly enriched. Table 2 lists these gene sets and their *p* values in their respective leukemia subtypes. Figure 4 shows the gene set enrichment plots for the two highest enriched gene sets.

**Table 2:** Highly enriched gene sets in specific combinations of signaling networks and leukemia subtypes, with the nominal *p* values produced by GSEA for these sets in their respective combinations

Subtype	Network	*p* value	Gene set
Hyperdiploid	Apoptosis	0.0083	NFKB1, RELA, BCL2, PPP3CA, PPP3CB, PPP3CC, IL3RA, TNF, BAD,
			PPP3R1, AKT3, AKT1, AKT2, CHUK, TNFRSF1A, CASP7, DFFA,
			IKBKB, IKBKG, CASP3, IL3, CASP10, CSF2RB
ETV6_RUNX1	Cell cycle	0.0151	TFDP1, TFDP2, E2F1, RBP3, E2F2, E2F3, CCND3, RBL1, PRB1, RBL2,
			CCNE1, CCNE2, CDK4, CDK6, CCND1, CCND2, CCNA1, CDK2, CCNA2
T-ALL	Apoptosis	0.0162	BIRC2, BIRC3, XIAP, BIRC7, CASP7, NFKB1, IL1RAP, TRADD, FASLG,
			RELA, CASP3, DFFA, FADD, CASP8, IL1R1, FAS
TCF3-PBX1	Apoptosis	0.0167	PRKACA, PRKACB, PRKACG, PRKAR1A, PRKAR1B, IL1RAP, FADD,
			PRKAR2A, PRKAR2B, PRKX, BAD, IL1A, IL1B, IL1R1, TNFRSF10B,
			TNFRSF10C, TNFRSF10D
Hypo	Apoptosis	0.0484	CAPN1, CAPN2, IRAK3, IRAK1, IRAK4, MAP3K14, BCL2, TP53,
			NGF, NTRK1
Ph	Cell cycle	0.088	BUB1, BUB3, CDKN2A, CDK4, CDK6, CCND1, GADD45A, GADD45B
			CCND2, CCND3, RB1, CDC45L, MCM7, MCM2, CDC25A, CDKN1A,
			MCM6, MCM5, MCM4, MCM3, CCNL1, LAT, CCNE1, CCNE2, CDK2,
			ORC3L, ORC5L, ORC4L, ORC2L, ORC1L, ORC6L, TP53, GADD45G,
			CDC2, CCNA2, CCNA1, CDKN1B, CDKN1C

We observe from Table 2 that apoptosis and cell cycle signaling networks are dominant in all gene sets that are highly enriched. This implies a fundamental role for these two networks in the listed subtypes. It also implies that these subtypes are either caused by or leading to a perturbation in their respective gene sets. Another noteworthy observation is that, although all the highly enriched gene sets belong to only two networks, there is little overlap between them. In apoptosis for instance, PPP3 genes are dominant in hyperdiploid, while BIRC genes are dominant in T-ALL, and PRKA genes are dominant in TCF3-PBX1. Additionally, from Fig. 4, we observe that, although apoptosis and cell cycle have the highest enriched gene sets for hyperdiploid and ETV6_RUNX1, respectively, their relations to their respective leukemia subtypes are not the same. All genes in the apoptosis set in hyperdiploid exhibit higher expression than in other subtypes, which implies up-regulation of these genes in hyperdiploid. On the other hand, most of the genes in the cell cycle set in ETV6_RUNX1 have lower expression than in other subtypes, which indicates down-regulation of these genes in ETV6_RUNX1.

## Conclusions

In this paper, we presented a novel method for computing edge probability in signaling networks. Our method uses gene coexpression as input and computes the edge probabilities so that reachability between edge terminals is as close as possible to their empirical values obtained from gene transcription levels. We used our method to compute edge probabilities for four KEGG signaling networks, using gene expression data for seven leukemia subtypes. We also used the computed edge probabilities to compute a centrality value for every gene in every leukemia subtype. We analyzed the interactions and genes with outstanding probability and centrality in specific subtypes. We also analyzed similarities and differences among these subtypes based on their edge probabilities. We performed gene set enrichment analysis on the set of edges with outstanding probabilities in each subtype to study the significance of the results. Our analysis provided evidence that links specific gene sets to specific leukemia subtypes, which makes them strong candidates for investigation in the scope of their respective subtypes.
